# *trans*-Dibromidobis[tris­(4-chloro­phen­yl)phosphine]palladium(II)

**DOI:** 10.1107/S1600536809045413

**Published:** 2009-11-14

**Authors:** Leo Kirsten, Gideon Steyl

**Affiliations:** a Department of Chemistry, University of the Free State, Bloemfontein 9300, South Africa

## Abstract

In the title compound, [PdBr_2_(C_18_H_12_Cl_3_P)_2_], the Pd^II^ ion is situated on a centre of symmetry and is coordinated by two Br anions [Pd—Br = 2.4252 (2) Å] and two P-donor ligands [Pd—P = 2.3317 (6) Å] in a slightly distorted square-planar geometry [P—Pd—Br = 86.589 (15)°].

## Related literature

The title compound is isostructural with the corresponding dichlorido complex, *trans*-[PdCl_2_{P(*p*-ClPh)_3_}_2_], see: Kolosova *et al.* (1986 ▶).
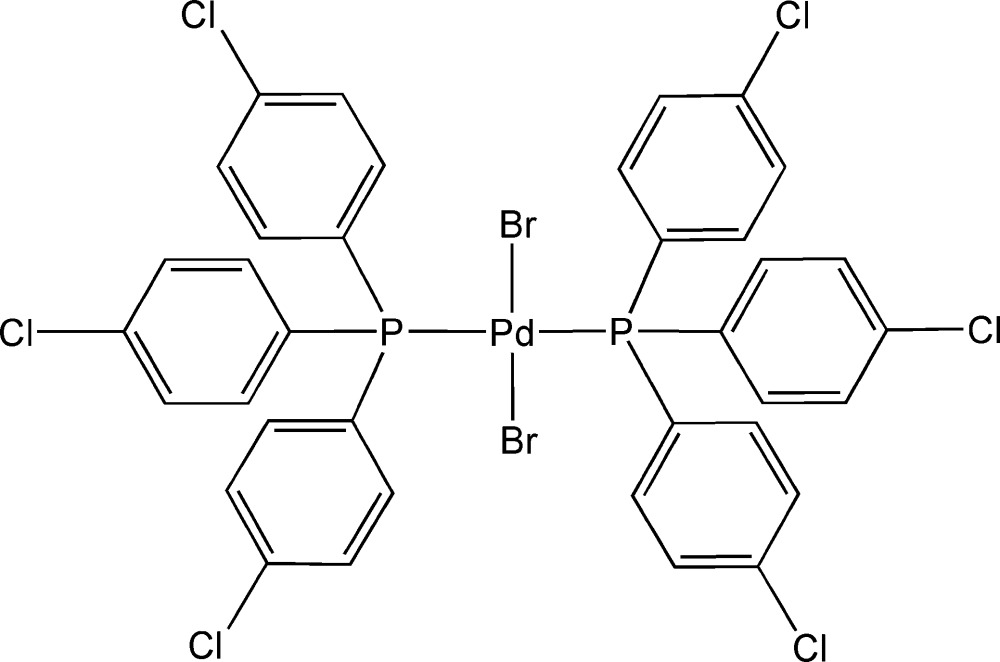


## Experimental

### 

#### Crystal data

[PdBr_2_(C_18_H_12_Cl_3_P)_2_]
*M* *_r_* = 997.41
Monoclinic, 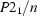

*a* = 10.3453 (3) Å
*b* = 17.4489 (6) Å
*c* = 10.6180 (4) Å
β = 103.385 (2)°
*V* = 1864.63 (11) Å^3^
*Z* = 2
Mo *K*α radiation
μ = 3.18 mm^−1^
*T* = 100 K
0.33 × 0.30 × 0.25 mm

#### Data collection

Bruker X8 APEXII 4K Kappa CCD diffractometer
Absorption correction: multi-scan (*SADABS*; Bruker, 1998 ▶) *T*_min_ = 0.378, *T*_max_ = 0.453
20106 measured reflections
4487 independent reflections
3916 reflections with *I* > 2σ(*I*)
*R*_int_ = 0.046

#### Refinement

*R*[*F* ^2^ > 2σ(*F* ^2^)] = 0.026
*wR*(*F* ^2^) = 0.060
*S* = 1.04
4487 reflections
214 parameters
H-atom parameters constrained
Δρ_max_ = 0.67 e Å^−3^
Δρ_min_ = −0.74 e Å^−3^

### 

Data collection: *APEX2* (Bruker, 2005 ▶); cell refinement: *SAINT-Plus* (Bruker, 2004 ▶); data reduction: *SAINT-Plus* and *XPREP* (Bruker, 2004 ▶); program(s) used to solve structure: *SHELXS97* (Sheldrick, 2008 ▶); program(s) used to refine structure: *SHELXL97* (Sheldrick, 2008 ▶); molecular graphics: *DIAMOND* (Brandenburg & Putz, 2006 ▶); software used to prepare material for publication: *SHELXL97*.

## Supplementary Material

Crystal structure: contains datablocks I, global. DOI: 10.1107/S1600536809045413/cv2637sup1.cif

Structure factors: contains datablocks I. DOI: 10.1107/S1600536809045413/cv2637Isup2.hkl

Additional supplementary materials:  crystallographic information; 3D view; checkCIF report

## Figures and Tables

**Fig. 1.**
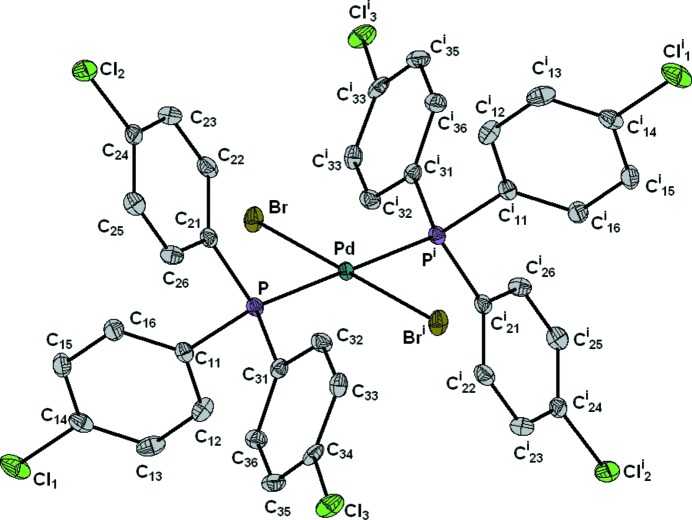
Representation of the title compound, showing the numbering scheme and displacement ellipsoids (50% probability). For the carbon rings, the first digit refers to ring number, second digit to atom in the ring. Hydrogen atoms omitted for clarity [symmetry code: (i) 1 - x,1 - y,1 - z].

## supplementary crystallographic information

### Comment

The title compound, (I), is one of a few literature structures containing a Pd centre coordinated by to Br atoms and two P donor ligands in a *trans* configuration. Our research is aimed at expanding the number of known structures of the aforementioned type and to finally generalize on the crystallization mode, if any, of these complexes.

The molecule of (I) (Fig. 1), is centrosymmetric with pairs of equivalent P donor ligands *trans* to one another in a slightly distorted square-planar geometry [P—Pd—Br 86.589 (15)°].

The corresponding chloro complex (Kolosova *et al.*, 1986) is iso-structural to (I) when comparing the geometrical parameters. The RMS error of 0.097 Å also indicate the iso-structurality of the two complexes (the title complex superimposed with the corresponding dichloro-palladium complex (Kolosova *et al.*, 1986) including all the atoms except the hydrogen atoms).

### Experimental

The title complex was synthesized by the addition of 2.2 equivalents of tris(4-Cl-phenyl)-phosphine (21 mg, 0.059 mmol) to [Pd(COD)Br_2_] (10 mg, 0.026 mmol) in 10 ml of dichloromethane while stirring for 5 minutes. Slow evaporation of the solvent resulted in orange crystals suitable for X-Ray diffraction (yield 74%, 19 mg).

### Refinement

All H atoms were positioned geometrically and allowed to ride on their parent atoms, with C—H = 0.95 Å and *U*_iso_(H) = 1.2*U*_eq_(C).

### Crystal data

**Table:**

[PdBr_2_(C_18_H_12_Cl_3_P)_2_]	*F*(000) = 976
*M**_r_* = 997.41	*D*_x_ = 1.776 Mg m^−^^3^
Monoclinic, *P*2_1_/*n*	Mo *K*α radiation, λ = 0.71073 Å
Hall symbol: -P 2yn	Cell parameters from 7624 reflections
*a* = 10.3453 (3) Å	θ = 2.3–28.4°
*b* = 17.4489 (6) Å	µ = 3.18 mm^−^^1^
*c* = 10.6180 (4) Å	*T* = 100 K
β = 103.385 (2)°	Cuboid, orange
*V* = 1864.63 (11) Å^3^	0.33 × 0.30 × 0.25 mm
*Z* = 2	

### Data collection

**Table:**

Bruker X8 APEXII 4K Kappa CCD diffractometer	4487 independent reflections
Radiation source: fine-focus sealed tube	3916 reflections with *I* > 2σ(*I*)
graphite	*R*_int_ = 0.046
Detector resolution: 512 pixels mm^-1^	θ_max_ = 28.0°, θ_min_ = 2.3°
φ and ω scans	*h* = −13→13
Absorption correction: multi-scan (*SADABS*; Bruker, 1998)	*k* = −20→23
*T*_min_ = 0.378, *T*_max_ = 0.453	*l* = −10→14
20106 measured reflections	

### Refinement

**Table:**

Refinement on *F*^2^	Primary atom site location: structure-invariant direct methods
Least-squares matrix: full	Secondary atom site location: difference Fourier map
*R*[*F*^2^ > 2σ(*F*^2^)] = 0.026	Hydrogen site location: riding model
*wR*(*F*^2^) = 0.060	H-atom parameters constrained
*S* = 1.04	*w* = 1/[σ^2^(*F*_o_^2^) + (0.0187*P*)^2^ + 1.3608*P*] where *P* = (*F*_o_^2^ + 2*F*_c_^2^)/3
4487 reflections	(Δ/σ)_max_ = 0.002
214 parameters	Δρ_max_ = 0.67 e Å^−^^3^
0 restraints	Δρ_min_ = −0.74 e Å^−^^3^

### Special details

**Table:**

Geometry. All e.s.d.'s (except the e.s.d. in the dihedral angle between two l.s. planes) are estimated using the full covariance matrix. The cell e.s.d.'s are taken into account individually in the estimation of e.s.d.'s in distances, angles and torsion angles; correlations between e.s.d.'s in cell parameters are only used when they are defined by crystal symmetry. An approximate (isotropic) treatment of cell e.s.d.'s is used for estimating e.s.d.'s involving l.s. planes.
Refinement. Refinement of *F*^2^ against ALL reflections. The weighted *R*-factor *wR* and goodness of fit *S* are based on *F*^2^, conventional *R*-factors *R* are based on *F*, with *F* set to zero for negative *F*^2^. The threshold expression of *F*^2^ > σ(*F*^2^) is used only for calculating *R*-factors(gt) *etc*. and is not relevant to the choice of reflections for refinement. *R*-factors based on *F*^2^ are statistically about twice as large as those based on *F*, and *R*- factors based on ALL data will be even larger.

### Fractional atomic coordinates and isotropic or equivalent isotropic displacement parameters (Å^2^)

**Table:**

	*x*	*y*	*z*	*U*_iso_*/*U*_eq_	
Pd	0.5000	0.5000	0.5000	0.01113 (6)	
Br	0.66195 (2)	0.551557 (14)	0.68290 (2)	0.01764 (7)	
P	0.54624 (5)	0.60598 (3)	0.38405 (5)	0.01106 (12)	
C11	0.7244 (2)	0.61782 (12)	0.4023 (2)	0.0121 (4)	
C12	0.7977 (2)	0.55569 (14)	0.3742 (2)	0.0181 (5)	
H12	0.7539	0.5087	0.3466	0.022*	
C13	0.9335 (2)	0.56171 (14)	0.3862 (2)	0.0202 (5)	
H13	0.9827	0.5200	0.3637	0.024*	
C14	0.9961 (2)	0.62964 (14)	0.4314 (2)	0.0167 (5)	
C15	0.9267 (2)	0.69103 (14)	0.4626 (2)	0.0180 (5)	
H15	0.9716	0.7371	0.4943	0.022*	
C16	0.7899 (2)	0.68506 (13)	0.4474 (2)	0.0166 (5)	
H16	0.7410	0.7274	0.4682	0.020*	
Cl1	1.16679 (6)	0.63849 (4)	0.44886 (6)	0.02709 (15)	
C21	0.4895 (2)	0.69919 (13)	0.4282 (2)	0.0127 (4)	
C22	0.4671 (2)	0.71256 (14)	0.5508 (2)	0.0165 (5)	
H22	0.4778	0.6720	0.6121	0.020*	
C23	0.4291 (2)	0.78460 (14)	0.5841 (2)	0.0193 (5)	
H23	0.4141	0.7936	0.6678	0.023*	
C24	0.4134 (2)	0.84297 (13)	0.4944 (2)	0.0158 (5)	
C25	0.4340 (2)	0.83135 (14)	0.3719 (2)	0.0169 (5)	
H25	0.4222	0.8721	0.3109	0.020*	
C26	0.4722 (2)	0.75940 (13)	0.3396 (2)	0.0160 (5)	
H26	0.4869	0.7509	0.2557	0.019*	
Cl2	0.36890 (6)	0.93429 (3)	0.53545 (6)	0.02335 (14)	
C31	0.4774 (2)	0.60521 (12)	0.2097 (2)	0.0123 (4)	
C32	0.3402 (2)	0.61279 (13)	0.1658 (2)	0.0151 (5)	
H32	0.2860	0.6153	0.2267	0.018*	
C33	0.2820 (2)	0.61675 (13)	0.0353 (2)	0.0154 (5)	
H33	0.1885	0.6221	0.0059	0.018*	
C34	0.3626 (2)	0.61276 (12)	−0.0525 (2)	0.0150 (5)	
C35	0.4977 (2)	0.60225 (13)	−0.0127 (2)	0.0170 (5)	
H35	0.5508	0.5975	−0.0743	0.020*	
C36	0.5555 (2)	0.59876 (13)	0.1195 (2)	0.0151 (5)	
H36	0.6488	0.5919	0.1483	0.018*	
Cl3	0.28975 (6)	0.62480 (3)	−0.21644 (5)	0.02222 (13)	

### Atomic displacement parameters (Å^2^)

**Table:**

	*U*^11^	*U*^22^	*U*^33^	*U*^12^	*U*^13^	*U*^23^
Pd	0.01157 (11)	0.01282 (13)	0.00824 (12)	−0.00270 (9)	0.00075 (8)	0.00108 (9)
Br	0.01929 (12)	0.01956 (13)	0.01117 (12)	−0.00653 (9)	−0.00241 (9)	0.00144 (9)
P	0.0110 (3)	0.0124 (3)	0.0096 (3)	−0.0017 (2)	0.0020 (2)	0.0013 (2)
C11	0.0124 (10)	0.0127 (11)	0.0109 (10)	−0.0004 (8)	0.0019 (8)	0.0035 (8)
C12	0.0186 (11)	0.0145 (12)	0.0192 (12)	0.0000 (9)	0.0002 (9)	−0.0033 (9)
C13	0.0218 (12)	0.0213 (13)	0.0168 (12)	0.0098 (10)	0.0033 (10)	−0.0025 (10)
C14	0.0118 (10)	0.0272 (13)	0.0112 (11)	0.0012 (10)	0.0027 (8)	0.0019 (9)
C15	0.0157 (11)	0.0157 (12)	0.0229 (13)	−0.0039 (9)	0.0051 (9)	−0.0008 (9)
C16	0.0153 (11)	0.0121 (11)	0.0237 (13)	0.0013 (9)	0.0069 (9)	0.0000 (9)
Cl1	0.0114 (3)	0.0469 (4)	0.0236 (3)	0.0021 (3)	0.0054 (2)	0.0013 (3)
C21	0.0094 (10)	0.0157 (12)	0.0126 (11)	−0.0022 (8)	0.0020 (8)	−0.0005 (9)
C22	0.0165 (11)	0.0195 (12)	0.0149 (11)	−0.0026 (9)	0.0062 (9)	0.0029 (9)
C23	0.0200 (12)	0.0221 (13)	0.0184 (12)	−0.0005 (10)	0.0099 (10)	−0.0033 (10)
C24	0.0105 (10)	0.0146 (12)	0.0235 (12)	−0.0017 (9)	0.0065 (9)	−0.0043 (9)
C25	0.0155 (11)	0.0181 (12)	0.0168 (11)	0.0004 (9)	0.0030 (9)	0.0033 (9)
C26	0.0176 (11)	0.0189 (12)	0.0124 (11)	0.0009 (9)	0.0054 (9)	0.0009 (9)
Cl2	0.0251 (3)	0.0184 (3)	0.0303 (3)	0.0016 (2)	0.0141 (3)	−0.0046 (2)
C31	0.0165 (11)	0.0090 (11)	0.0110 (10)	−0.0012 (8)	0.0022 (8)	0.0011 (8)
C32	0.0166 (11)	0.0169 (12)	0.0125 (11)	−0.0008 (9)	0.0050 (9)	0.0017 (9)
C33	0.0152 (11)	0.0128 (12)	0.0164 (11)	−0.0010 (9)	0.0002 (9)	0.0021 (9)
C34	0.0247 (12)	0.0098 (11)	0.0082 (10)	−0.0006 (9)	−0.0006 (9)	−0.0015 (8)
C35	0.0237 (12)	0.0161 (12)	0.0131 (11)	0.0016 (9)	0.0079 (9)	−0.0009 (9)
C36	0.0156 (11)	0.0151 (12)	0.0158 (11)	0.0016 (9)	0.0058 (9)	0.0005 (9)
Cl3	0.0325 (3)	0.0227 (3)	0.0093 (3)	0.0015 (3)	0.0003 (2)	−0.0014 (2)

### Geometric parameters (Å, °)

**Table:**

Pd—P^i^	2.3317 (6)	C22—C23	1.387 (3)
Pd—P	2.3317 (6)	C22—H22	0.9500
Pd—Br	2.4252 (2)	C23—C24	1.378 (3)
Pd—Br^i^	2.4252 (2)	C23—H23	0.9500
P—C11	1.820 (2)	C24—C25	1.381 (3)
P—C31	1.823 (2)	C24—Cl2	1.742 (2)
P—C21	1.827 (2)	C25—C26	1.383 (3)
C11—C16	1.384 (3)	C25—H25	0.9500
C11—C12	1.394 (3)	C26—H26	0.9500
C12—C13	1.386 (3)	C31—C36	1.393 (3)
C12—H12	0.9500	C31—C32	1.394 (3)
C13—C14	1.382 (3)	C32—C33	1.379 (3)
C13—H13	0.9500	C32—H32	0.9500
C14—C15	1.372 (3)	C33—C34	1.389 (3)
C14—Cl1	1.739 (2)	C33—H33	0.9500
C15—C16	1.391 (3)	C34—C35	1.376 (3)
C15—H15	0.9500	C34—Cl3	1.742 (2)
C16—H16	0.9500	C35—C36	1.394 (3)
C21—C26	1.394 (3)	C35—H35	0.9500
C21—C22	1.395 (3)	C36—H36	0.9500
			
P^i^—Pd—P	180.000 (17)	C23—C22—C21	120.5 (2)
P^i^—Pd—Br	93.411 (15)	C23—C22—H22	119.7
P—Pd—Br	86.589 (15)	C21—C22—H22	119.7
P^i^—Pd—Br^i^	86.589 (15)	C24—C23—C22	119.3 (2)
P—Pd—Br^i^	93.411 (15)	C24—C23—H23	120.4
Br—Pd—Br^i^	180.000 (10)	C22—C23—H23	120.4
C11—P—C31	104.88 (10)	C23—C24—C25	121.6 (2)
C11—P—C21	104.43 (10)	C23—C24—Cl2	119.94 (17)
C31—P—C21	101.18 (10)	C25—C24—Cl2	118.50 (18)
C11—P—Pd	111.12 (7)	C24—C25—C26	118.8 (2)
C31—P—Pd	116.79 (7)	C24—C25—H25	120.6
C21—P—Pd	116.95 (7)	C26—C25—H25	120.6
C16—C11—C12	119.1 (2)	C25—C26—C21	121.1 (2)
C16—C11—P	122.37 (17)	C25—C26—H26	119.5
C12—C11—P	118.44 (17)	C21—C26—H26	119.5
C13—C12—C11	120.8 (2)	C36—C31—C32	119.1 (2)
C13—C12—H12	119.6	C36—C31—P	123.10 (17)
C11—C12—H12	119.6	C32—C31—P	117.81 (16)
C14—C13—C12	118.7 (2)	C33—C32—C31	121.0 (2)
C14—C13—H13	120.6	C33—C32—H32	119.5
C12—C13—H13	120.6	C31—C32—H32	119.5
C15—C14—C13	121.6 (2)	C32—C33—C34	118.8 (2)
C15—C14—Cl1	118.69 (18)	C32—C33—H33	120.6
C13—C14—Cl1	119.69 (18)	C34—C33—H33	120.6
C14—C15—C16	119.3 (2)	C35—C34—C33	121.7 (2)
C14—C15—H15	120.4	C35—C34—Cl3	119.80 (17)
C16—C15—H15	120.4	C33—C34—Cl3	118.46 (18)
C11—C16—C15	120.5 (2)	C34—C35—C36	118.9 (2)
C11—C16—H16	119.8	C34—C35—H35	120.5
C15—C16—H16	119.8	C36—C35—H35	120.5
C26—C21—C22	118.8 (2)	C31—C36—C35	120.4 (2)
C26—C21—P	119.79 (16)	C31—C36—H36	119.8
C22—C21—P	121.43 (17)	C35—C36—H36	119.8
			
Br—Pd—P—C11	49.81 (8)	Pd—P—C21—C22	23.8 (2)
Br^i^—Pd—P—C11	−130.19 (8)	C26—C21—C22—C23	−0.4 (3)
Br—Pd—P—C31	170.03 (8)	P—C21—C22—C23	177.41 (17)
Br^i^—Pd—P—C31	−9.97 (8)	C21—C22—C23—C24	0.2 (3)
Br—Pd—P—C21	−69.94 (8)	C22—C23—C24—C25	0.3 (3)
Br^i^—Pd—P—C21	110.06 (8)	C22—C23—C24—Cl2	−178.76 (17)
C31—P—C11—C16	109.0 (2)	C23—C24—C25—C26	−0.4 (3)
C21—P—C11—C16	3.0 (2)	Cl2—C24—C25—C26	178.59 (17)
Pd—P—C11—C16	−123.98 (18)	C24—C25—C26—C21	0.2 (3)
C31—P—C11—C12	−73.49 (19)	C22—C21—C26—C25	0.2 (3)
C21—P—C11—C12	−179.49 (17)	P—C21—C26—C25	−177.63 (17)
Pd—P—C11—C12	53.56 (19)	C11—P—C31—C36	11.9 (2)
C16—C11—C12—C13	−2.6 (3)	C21—P—C31—C36	120.28 (19)
P—C11—C12—C13	179.82 (18)	Pd—P—C31—C36	−111.59 (18)
C11—C12—C13—C14	2.5 (4)	C11—P—C31—C32	−166.98 (17)
C12—C13—C14—C15	−0.9 (4)	C21—P—C31—C32	−58.60 (19)
C12—C13—C14—Cl1	179.37 (18)	Pd—P—C31—C32	69.53 (18)
C13—C14—C15—C16	−0.6 (4)	C36—C31—C32—C33	−2.4 (3)
Cl1—C14—C15—C16	179.15 (18)	P—C31—C32—C33	176.56 (18)
C12—C11—C16—C15	1.0 (3)	C31—C32—C33—C34	0.2 (3)
P—C11—C16—C15	178.56 (17)	C32—C33—C34—C35	2.4 (3)
C14—C15—C16—C11	0.5 (3)	C32—C33—C34—Cl3	−175.38 (17)
C11—P—C21—C26	78.41 (19)	C33—C34—C35—C36	−2.7 (3)
C31—P—C21—C26	−30.3 (2)	Cl3—C34—C35—C36	175.00 (18)
Pd—P—C21—C26	−158.34 (15)	C32—C31—C36—C35	2.0 (3)
C11—P—C21—C22	−99.40 (19)	P—C31—C36—C35	−176.87 (17)
C31—P—C21—C22	151.87 (18)	C34—C35—C36—C31	0.5 (3)

Symmetry codes: (i) −*x*+1, −*y*+1, −*z*+1.
